# Knowledge and Attitudes of First Aid and Basic Life Support Among Public School Teachers in Qassim, Saudi Arabia

**DOI:** 10.7759/cureus.42955

**Published:** 2023-08-04

**Authors:** Yazeed S Alahmed, Haytham S Alzeadi, Anfal K Alghumayzi, Leen A Almarshad, Abdulmajeed S Alharbi, Abdulelah S Alharbi

**Affiliations:** 1 Pediatrics, Qassim University, Buraidah, SAU; 2 Medicine, Qassim University, Buraidah, SAU; 3 Medicine, Qassim University, Unaizah, SAU

**Keywords:** cardiopulmonary resuscitation (cpr), skill, basic life support (bls), saudi arabia, schools, teachers, first aid, assessment, attitude, knowledge

## Abstract

Objective: This study aims to address the knowledge gap in first aid and basic life support (BLS) among teachers, expand the targeted schools to elementary and intermediate schools for boys and girls, and develop clear, focused recommendations. Furthermore, to assess the knowledge, skills, and attitude of BLS among schoolteachers in Qassim, Saudi Arabia.

Methods: This cross-sectional study was conducted in the school year of 2022 to 2023. In Buraidah city and the Uyun AlJiwa and Asyah governorates of Qassim, there are a total of 906 elementary and intermediate schools employing 12,057 teachers (5447 males and 6610 females). A stratified random sampling method was used with a self-administered Arabic questionnaire. This questionnaire included multiple parts (sociodemographic data, previous training status, knowledge and skill of BLS, and assessment of the following: attitude to learn and practice CPR; barriers to performing CPR; the presence or lack of previous resuscitation experience in BLS). Data were analyzed using SPSS Statistics version 25 (IBM Corp., Armonk, NY, USA). Categorical variables were described by frequency and percentage, while continuous variables were described by mean ± SD. A normality test showed that the total knowledge and skills scale was not normally distributed. The Mann-Whitney and Kruskal-Wallis tests were used to compare the mean knowledge and skills scale across variables. The accepted level of significance was below 0.05 (p <0.05).

Results: Our study included 482 participants. Only 19.5% (94) had previous CPR training, and 80.9% (76) were trained more than two years prior to this study's data collection. The main reason for participants' fears of applying BLS was the lack of proper knowledge and skills (48.1%). The majority of the teachers, i.e., 71.0% (342), wanted more training in CPR, and 41.1% (198) thought CPR training should be mandatory at school. We found no statistical relationship between attitude toward training and the city or differences in knowledge and skills scores due to the difference in sociodemographic characteristics. Also, we found no statistical relationship between the question *'Did you observe CPR on a collapsed patient?' *and the city, meaning that the observation of CPR on collapsed patients is independent of the respondent's school location. Significant differences in skills scores were found between those who had CPR observation and those who did not (p = 0.014), in knowledge scores between those who had previous CPR training and those who did not (p = 0.034), and in skills scores between those who had previous CPR training and those who did not (p <0.001). We found no significant differences in knowledge and skills scores according to the place of previous CPR training (p = 0.163 and p = 0.695, respectively).

Conclusion: This study reveals that knowledge and skills in BLS among schoolteachers need to be improved. For this reason, we emphasize the inclusion of International Liaison Committee on Resuscitation (ILCOR) recommendations in the curriculum and that they are made periodic and mandatory for teachers. Especially as we found teachers to have a positive attitude and were willing to train and help.

## Introduction

Children spend considerable time on school premises away from their parents and guardians. For these children, school is a ‘home away from home’ and is meant to be a ‘safe haven’ that provides them with the necessary healthy environment to learn, play, and interact with other students [1]. As they are exposed to various types of epidemiological factors in school, medical emergencies may occur unexpectedly. Thus, instant and appropriate first aid help must be available [1,2].

We define first aid as the assessments and interventions that can be performed by a bystander (or by the victim) with minimal or no medical equipment. A first aid provider is defined as someone with formal training in first aid, emergency care, or medicine who provides first aid [3]. Cardiac arrest (CA) is a life-threatening medical emergency that happens outside hospital settings in 70% of cases and has a high mortality rate [2]. The cardiovascular disease mortality rate is 37% in Saudi Arabia [4], 75% of sudden deaths among young athletes, and 5% to 10% of all deaths in children between the ages of five and 19 are caused by sudden cardiac death [5], although the ability to perform basic life support (BLS) can change the outcome from death to survival [6]. Subsequently, having a trained rescuer who is ready, willing, and able to act is considered to be the most important determinant of surviving a sudden cardiac arrest [7]. Even though CPR has been proven to be effective, only about one in every three out-of-hospital victims who witness cardiopulmonary arrest (CPA) receives lifesaving assistance from a bystander [8]. Initially, BLS skills were aimed at healthcare workers. Subsequently, the survival rate for every minute delay in the initiation of CPR has been shown to decrease by 7% to 10%. Bystander-initiated CPR with the early use of automated external defibrillators (AEDs) may help save lives. Basic life support, including CPR, has now evolved into a skill taught to the common people. The early initiation of bystander CPR in cases of witnessed cardiac arrest has been shown to improve survival outcomes by approximately 50% [1]. The 2020 American Heart Association (AHA) guidelines simplify resuscitation and re-emphasize the importance of early initiation of CPR by lay rescuers by providing rapid and deep compressions with minimal interruptions immediately after the shock given by the AED, as it is considered the "chain of survival" [9].

In 1995, the WHO launched the Global School Health Initiative, aiming to increase the number of health-promoting schools. While the definition may vary depending on the needs and conditions, a health-promoting school can be described as constantly strengthening its capacity as a healthy setting for living, learning, and working [10]. Additionally, the International Liaison Committee on Resuscitation (ILCOR) strongly recommends the inclusion of BLS in the school curriculum [11]. Recently, it has been established in Saudi Arabia by the Ministry of Education that every school must assign a health guide (health educator) with specific qualifications that include having an advanced first aid certification. The roles of the health guide comprise creating a healthy environment for the students, monitoring their health conditions, and managing first aid for emergencies before the arrival of paramedics [12].

Teachers are the primary people who can assist students and provide first aid, preventing fatal complications [13]. For this reason, this study aims to address the knowledge gap in first aid and BLS among teachers, expand the targeted schools to elementary and intermediate schools for boys and girls, and develop clear, focused recommendations. Furthermore, it also assesses the knowledge, skills, and attitudes of BLS among school teachers in Qassim, Saudi Arabia.

## Materials and methods

This cross-sectional study was conducted in the 2022 to 2023 school year. There are a total of 906 elementary and intermediate schools in Buraidah city and the Uyun AlJiwa and Asyah governorates of Qassim (376 boys schools and 530 girls schools) with 12,057 teachers (5447 males and 6610 females). A stratified random sampling was used with a self-administered Arabic-validated questionnaire [14]. This questionnaire included multiple parts (sociodemographic data, previous training status, knowledge and skill of BLS, questions to assess the attitude toward learning and practicing CPR, gauging the barriers to performing CPR, and whether participants had previous resuscitation experience in BLS or not).

Data were analyzed using SPSS Statistics version 25 (IBM Corp., Armonk, NY, USA). Categorical variables were described by frequency and percentage, while continuous variables were described by mean ± SD. For the knowledge and skills questions, the true response was given 1, and the wrong answer received 0. A normality test showed that the total knowledge and skills scale was not normally distributed. Mann-Whitney and Kruskal-Wallis tests were used to compare the mean knowledge and skills scale across variables. The accepted level of significance was below 0.05 (p <0.05).

Oral and written informed consent was obtained from all participants. This project was approved by the Committee of Research Ethics, Deanship of Scientific Research, Qassim University (approval no. 23-21-05).

## Results

Demographic characteristics of schoolteachers

Our study included 482 participants. Almost all of them (481, 99.8%) are Saudi; 384 (79.7%) teach at schools in Buraidah, 51 (10.6%) at schools in Uyun AlJiwaa, and 47 (9.8%) at schools in Asyah. Also, 58.9% are elementary school teachers, and 41.1% are intermediate school teachers. The majority have incomes between 13001 and 16000 Suadi riyal (SAR) and 10001 and 13000 SAR (34.0% to 32.6%), respectively. Most of those who answered the questionnaire (69.9%) reported that their school has someone responsible for dealing with emergencies (Table 1).

**Table 1:** Demographic characteristics of schoolteachers

Questionnaire	Multiple choice answers	N (%)	Total N (%)
At which school level (school type) do you work?	Elementary	284 (58.9)	482 (100)
	Intermediate	198 (41.1)	
Location (city) of your school	Buraidah	384 (79.7)	482 (100)
	Uyun AlJiwa	51 (10.6)	
	Asyah	47 (9.7)	
Gender	Male	221 (45.9)	482 (100)
	Female	261 (54.1)	
Nationality	Saudi	481 (99.8)	482 (100)
	Non-Saudi	1 (0.2)	
Monthly Income	4000 to 7000	21 (4.4)	482 (100)
	7001 to 10000	48 (9.9)	
	10001 to 13000	157 (32.6)	
	13001 to 16000	164 (34.0)	
	More Than 16000	92 (19.1)	
Is there a person responsible for dealing with emergencies in your school?	Yes	337 (69.9)	482 (100)
	No	66 (13.7)	
	I don't know	79 (16.4)	

Medical background of the primary and emergency care provider

The results indicate that 42.1% of respondents reported that one of the school staff with prior training should be made responsible for providing primary and emergency care; 22.8% did not know who should be made responsible for emergency care; 21.4% said a person from outside the school should be in charge; and 13.7% said a teacher with prior training should be made the emergency care provider. The existence of a competent person responsible for primary and emergency health care is important, according to the respondents (94.6%). More than half the respondents (53.5%) said they did not have an automated external defibrillator (AED) at the school, and 44.4% did not know if they had one. The majority (72.4%) of the respondents stated that the presence of an AED is important (Table 2).

**Table 2:** Medical background of the primary and emergency care provider AED: Automated external defibrillator

Questionnaire	Multiple choice answers	N (%)	Total N (%)
Who should be responsible for providing primary and emergency care?	A person from outside the school	103 (21.4)	482 (100)
	A teacher with prior training	66 (13.7)	
	One of the school staff with prior training	203 (42.1)	
	I don't know	110 (22.8)	
The existence of a competent person responsible for primary and emergency health care is...	Very Important	415 (86.1)	482 (100)
	Somewhat important	41 (8.5)	
	Neutral	22 (4.6)	
	Not important	3 (0.6)	
	Not important at all	1 (0.2)	
Is there an automated external defibrillator (AED) in your school?	Yes	10 (2.1)	482 (100)
	No	258 (53.5)	
	I don't know	214 (44.4)	
Do you think having this device (the AED) is important?	Very important	236 (49.0)	482 (100)
	Somewhat important	113 (23.4)	
	Neutral	118 (24.5)	
	Not important	14 (2.9)	
	Not important at all	1 (0.2)	

Training status of schoolteachers

**Figure 1:**
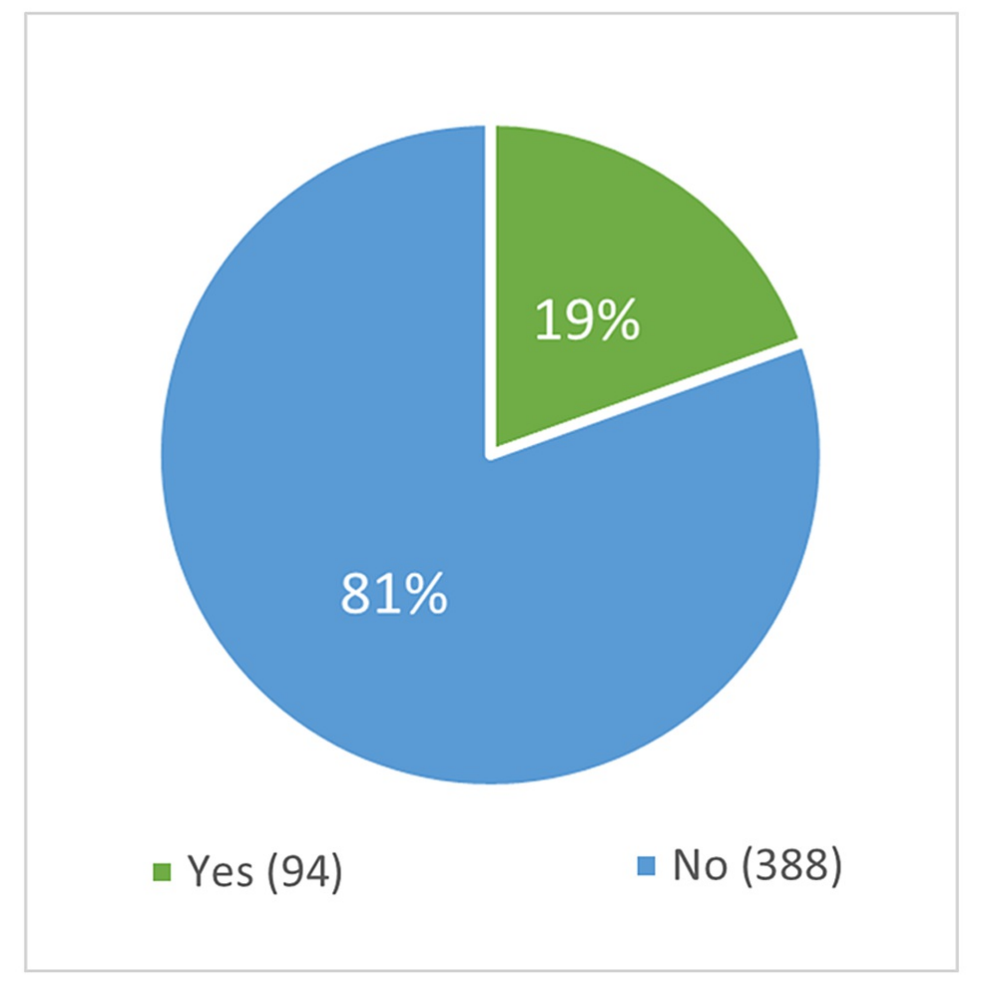
Proportion of participants with and without previous CPR training Total number of participants = 482

**Figure 2:**
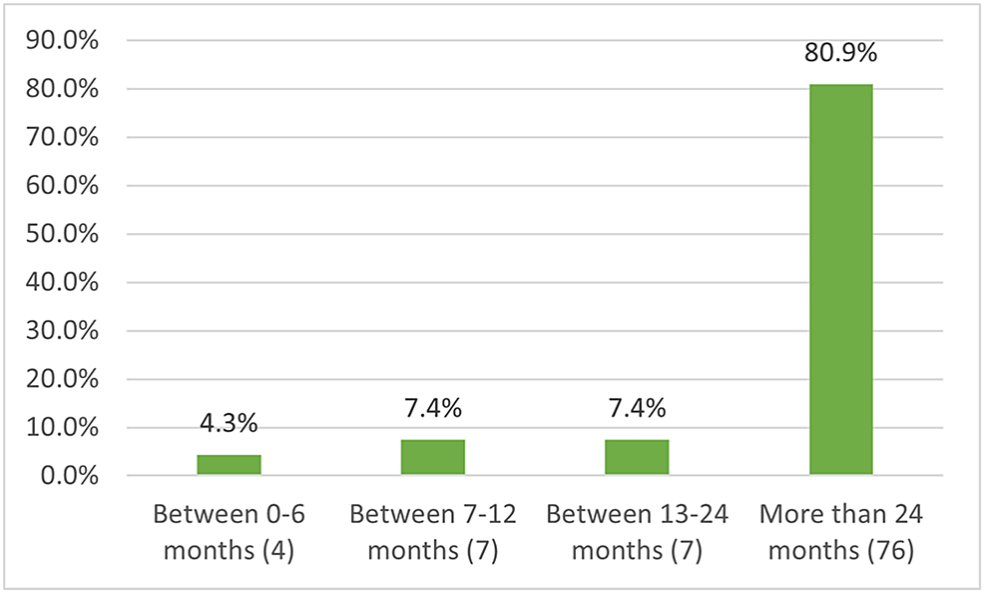
Time since previous CPR training Total number of participants = 482

**Figure 3:**
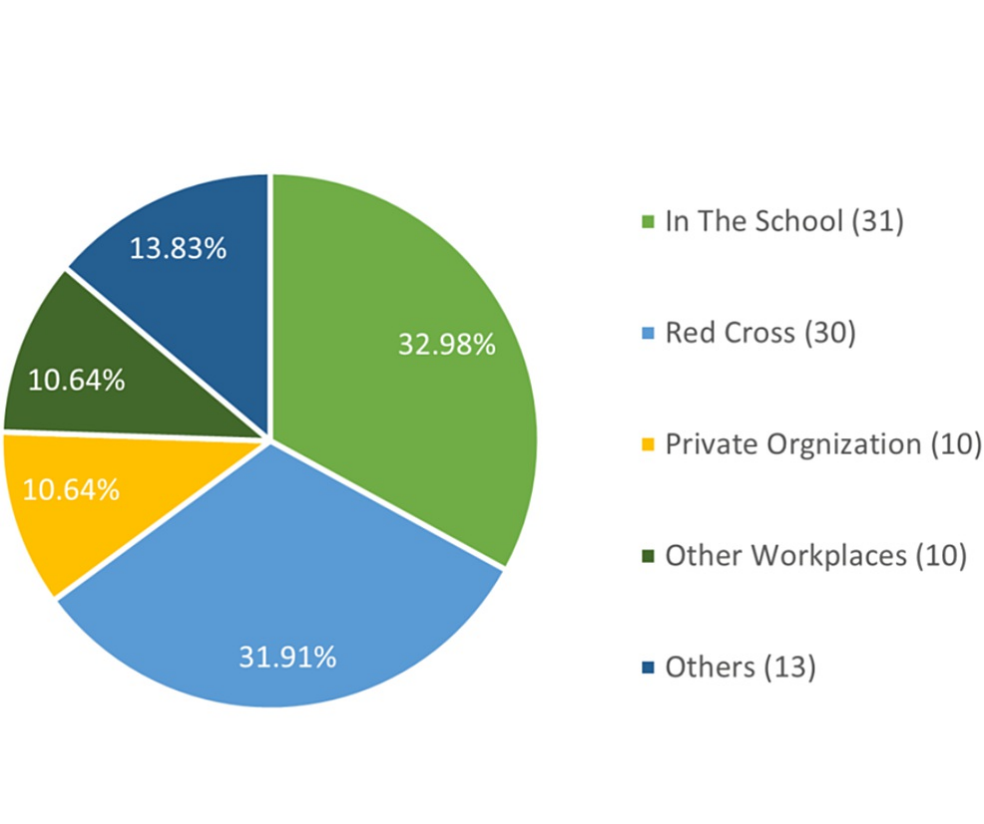
Where and how the respondents received CPR training Total number of participants = 482

Out of the 482 participants, only 19.5% (94) had previous CPR training (Figure 1), and 80.9% (76) of them got their training more than two years before this study's data collection (Figure 2). Of the 94 with CPR training, 32.98% (31) received training in school, and 31.9% (30) were trained by the Red Cross (Figure 3).

Knowledge assessment among schoolteachers

The highest proportion (87.3%) of correct responses was to the question,* 'What is the correct emergency phone number?'*, and the lowest proportion (11.2%) of correct responses was to the question, *'How deep and how fast would you perform chest compressions?'* (Table 3).

**Table 3:** Knowledge assessment questions Total number of participants = 482 AED: Automated external defibrillator

Questionnaire	Type of response	Buraidah	Uyon AlJiwa	Asyah	Total N (%)
What is the correct emergency phone number?	Correct response n (%)	340 (88.5)	48 (94.1)	33 (70.2)	421 (87.3)
	Incorrect response n (%)	44 (11.5)	3 (5.9)	14 (29.8)	61 (12.7)
You are alone and come across an apparently lifeless adult person. What do you do?	Correct response n (%)	213 (55.5)	32 (62.7)	21 (44.7)	266 (55.2)
	Incorrect response n (%)	171 (44.5)	19 (37.3)	26 (55.3)	216 (44.8)
The patient is breathing without response to verbal stimuli. What do you do?	Correct response n (%)	50 (75.8)	9 (13.6)	7 (10.6)	66 (13.7)
	Incorrect response n (%)	334 (87.0)	42 (82.4)	40 (85.1)	416 (86.3)
You decide to perform CPR. Which of the following combinations of chest compressions and ventilations would you choose?	Correct response n (%)	155 (40.4)	21 (41.2)	21 (41.2)	197 (40.9)
	Incorrect response n (%)	229 (59.6)	30 (58.8)	26 (55.3)	285 (59.1)
How deep and how fast would you perform chest compressions?	Correct response n (%)	45 (11.7)	6 (11.8)	4 (8.5)	55 (11.2)
	Incorrect response n (%)	339 (88.3)	45 (88.2)	43 (91.5)	427 (88.6)
What is the purpose of an automated external defibrillator (AED)?	Correct response n (%)	64 (16.7)	12 (23.5)	8 (17.0)	84 (17.4)
	Incorrect response n (%)	320 (83.3)	39 (76.5)	39 (83.0)	398 (82.6)
Who is allowed to use an AED?	Correct response n (%)	224 (58.3)	30 (58.8)	31 (66.0)	285 (59.1)
	Incorrect response n (%)	160 (41.7)	21 (41.2)	16 (34.0)	197 (40.9)

Skills assessment among schoolteachers 

The highest proportion (68.5%) of correct responses was to the question, *'Does the respondent kneel next to the torso?'*, and the question, *'What is the frequency of chest compressions?'* received the lowest proportion (14.3%) of correct responses. In addition, we found no statistical relationship between attitudes toward training and the city. In other words, attitudes toward training are independent of the respondent's school location. Also, with respect to the association between sociodemographic characteristics and the knowledge and skills assessment among our participants, we found that there are no statistical differences in knowledge and skills scores due to the difference in all variables (gender, stage of school, age, income, and city). In other words, all demographic variables do not affect the level of knowledge and skills of the respondents (p >0.05 for all variables) (Table 4).

**Table 4:** Skills assessment questions Total number of participants = 482

Questionnaire	Type of response	Buraidah	Uyon AlJiwa	Asyah	Total N (%)
Does the respondent kneel next to the torso?	Correct response n (%)	259 (67.4)	36 (70.6)	35 (74.5)	330 (68.5)
	Incorrect response n (%)	125 (32.6)	15 (29.4)	12 (25.5)	152 (31.5)
How is the hand-placement on the torso?	Correct response n (%)	240 (62.5)	27 (52.9)	25 (53.2)	292 (60.6)
	Incorrect response n (%)	144 (37.5)	24 (47.1)	22 (53.2)	190 (39.4)
What is the chest compression frequency?	Correct response n (%)	48 (12.5)	12 (23.5)	9 (19.1)	69 (14.3)
	Incorrect response n (%)	336 (87.5)	39 (76.5)	38 (80.9)	413 (85.7)

Attitude toward training among schoolteachers 

The main reasons mentioned for no previous CPR training were "not sure where to attend a course" (34.0%) and "little time" (25.0%). In the opinion of the participants, the main reason people fear applying BLS is the lack of proper knowledge and skills (48.1%). The majority of the teachers (71.0%) wanted more training in CPR, and 43.6% wanted to take a CPR course as they "wish to avoid unnecessary death". The majority of the teachers (77.2%) wanted free CPR courses. Around 41.1% think CPR training should be mandatory in schools, and 33.0% think it should be optional. About 67.8% said CPR is not already part of the educational curriculum, and only 23.8% of participants think CPR training should be required for teacher certification. Also, we found no statistical relationship between attitudes toward training and the city. In other words, attitudes toward training are independent of the respondent's school location (Table 5).

**Table 5:** Questions assessing attitude toward training Total number of participants = 482

Questionnaire	Multiple choice answers	Buraidah, N (%)	Uyon AlJiwa, N (%)	Asyah, N (%)	Total N (%)	p-value
If you had no previous CPR training, what is the reason?	Little interest	30 (78.9)	4 (10.5)	4 (10.5)	38 (9.8)	0.925
	Little time	79 (81.4)	8 (8.2)	10 (10.3)	97 (25.0)	
	Not sure where to attend a course	108 (81.8)	12 (9.1)	12 (9.1)	132 (34.0)	
	Costs	8 (80.0)	2 (20.0)	0 (0.0)	10 (3.6)	
	No answer	95 (84.1)	8 (7.1)	10 (8.8)	113 (29.1)	
Which reason do you think makes people afraid to apply BLS to victims?	Afraid of contagious diseases through mouth-to-mouth resuscitation	40 (81.6)	4 (8.2)	5 (10.2)	49 (10.2)	0.848
	Causing potential harm to the person in need	63 (79.7)	8 (10.1)	8 (10.1)	79 (16.4)	
	Afraid of legal consequences	53 (73.6)	11 (15.3)	8 (11.1)	72 (14.9)	
	Emotional factors	32 (86.5)	2 (5.4)	3 (8.1)	37 (7.7)	
	Lack of proper knowledge and skills	185 (79.7)	26 (11.2)	21 (9.1)	232 (48.1)	
	Other reasons	11 (84.6)	0 (0.0)	2 (15.4)	13 (2.7)	
Do you want more training?	Yes, strongly agree	117 (75.5)	18 (11.6)	20 (12.9)	155 (32.2)	0.261
	Yes	155 (82.9)	20 (10.7)	12 (6.4)	187 (38.8)	
	Nuteral	56 (81.2)	6 (8.7)	7 (10.1)	69 (14.3)	
	No interest	35 (72.9)	5 (10.4)	8 (16.7)	48 (10.0)	
	No interest at all	21 (91.3)	2 (8.7)	0 (0.0)	23 (4.8)	
If you want more CPR training, what is the reason?	Heart disease within the family	37 (80.4)	6 (13.0)	3 (6.5)	46 (9.5)	0.434
	Wish to avoid unnecessary death	173 (82.4)	15 (7.1)	22 (10.5)	210 (43.6)	
	Other reason or no answer	47 (73.4)	10 (15.6)	7 (10.9)	64 (13.3)	
	No answer	127 (78.4)	20 (12.3)	15 (9.3)	162 (33.6)	
Would you be willing to take a free CPR course if it were offered?	Yes, strongly agree	131 (77.1)	21 (12.4)	18 (10.6)	170 (35.3)	0.78
	Yes	164 (81.2)	19 (9.4)	19 (9.4)	202 (41.9)	
	Neutral	49 (81.7)	4 (6.7)	7 (11.7)	60 (12.5)	
	No Interest	25 (83.3)	3 (10.0)	2 (6.7)	30 (6.2)	
	No Interest at all	15 (75.0)	4 (20.0)	1 (5.0)	20 (4.2)	
Do you think CPR training should be mandatory?	Yes, in schools	149 (75.3)	26 (13.1)	23 (11.6)	198 (41.1)	0.6
	Yes, to obtain a driver's license	26 (86.7)	2 (6.7)	2 (6.7)	30 (6.2)	
	Yes, training should be mandatory in every job	78 (82.1)	8 (8.4)	9 (9.5)	95 (19.7)	
	No, CPR training should be optional	131 (82.4)	15 (9.4)	13 (8.2)	159 (33.0)	
Is CPR already part of the educational curriculum?	Yes	119 (76.8)	16 (10.3)	20 (12.9)	155 (32.2)	0.275
	No	265 (81.0)	35 (10.7)	27 (8.3)	327 (67.8)	
Do you think CPR training should be a requirement for teacher certification?	Yes, strongly agree	42 (71.2)	11 (18.6)	6 (10.2)	59 (12.2)	0.187
	Yes	44 (78.6)	7 (12.5)	5 (8.9)	56 (11.6)	
	Neutral	169 (78.2)	24 (11.1)	23 (10.6)	216 (44.8)	
	Somewhat disinterested	62 (89.9)	1 (1.4)	6 (8.7)	69 (14.3)	
	Disinterested overall	66 (81.5)	8 (9.9)	7 (8.6)	81 (16.8)	

Resuscitation experiences among schoolteachers 

Out of the 482 participants, 194 (40.25%) observed CPR on a collapsed patient, and only 24 (4.98%) had participated in CPR before. In addition, there is no statistical relationship between the question*, 'Did you observe CPR on a collapsed patient?'* and the city. In other words, the observation of CPR on collapsed patients is independent of the respondent's school location (Table 6). Also, regarding the association between resuscitation experience, knowledge, and skills assessment, we found a significant difference in skills score between those who had observed CPR (1.55 ± 0.795) and those who had not (1.35 ± 0.826) (p = 0.014).

**Table 6:** Questions on resuscitation experiences Total number of participants = 482

Questionnaire	Response	Buraidah, N (%)	Uyon AlJiwa, N (%)	Asyah, N (%)	Total, N (%)	p-value
Did you observe CPR on a collapsed patient?	Yes	146 (38.02%)	23 (45.10%)	25 (53.19%)	194 (40.25%)	0.102
	No	238 (61.98%)	28 (54.90%)	22 (46.81%)	288 (59.75%)	
Did you participate in CPR before?	Yes	17 (4.43%)	3 (5.88%)	4 (8.51%)	24 (4.98%)	0.455
	No	367 (95.57%)	48 (94.12%)	43 (91.49%)	458 (95.02%)	

Association between the place of CPR training, knowledge, and skills assessment among schoolteachers

There is a significant difference in knowledge score between those who had previous CPR training (3.09±1.232) and those who didn't (2.79±1.101) (p = 0.034). There is also a significant difference in skills score between those who had previous CPR training (1.72±0.678) and those who didn't (1.36±0.835) (p <0.001). There are no significant differences in knowledge and skills scores according to the place of previous CPR training (p = 0.163, p = 0.695, respectively) (Table 7). Additionally, we found that none of the participants correctly answered all the knowledge questions, while 31 (6.4%) of the participants correctly answered all the skills questions; 8 of them (25.8%) had previous CPR training as opposed to 23 (74.2%) who didn't.

**Table 7:** Association between the place of CPR training, knowledge, and skills assessment

Variables		Knowledge			Skills		
		Mean Rank	Mean±SD	p-value	Mean Rank	Mean±SD	p-value
Previous CPR training	Yes	267.9	3.09±1.232	0.034	287.5	1.72±0.678	0.000
	No	235.1	2.79±1.101		230.4	1.36±0.835	
Place of CPR training	In the school	38.90	2.68±1.222	0.163	42.98	1.58±0.62	0.695
	Red Cross	50.88	3.27±1.015		48.03	1.77±0.626	
	Private organization	55.20	3.4±1.35		47.90	1.7±0.823	
	Other work	58.90	3.5±1.434		52.60	1.9±0.738	
	Others	45.50	3.08±1.382		52.81	1.85±0.8	

## Discussion

The knowledge and skills among schoolteachers are inadequate regarding BLS techniques, specifically CPR. Despite the effectiveness of early initiation of bystander CPR in improving survival outcomes [1], our assessment showed that most participants (80.5%) had not received previous CPR training, and the remaining 20% could not answer all the questions correctly despite receiving prior training. Consequentially, this was reflected in the degree of participants' knowledge, as the highest proportion of correct responses were to general knowledge questions such as 'What is the correct emergency phone number?' and 'Who is allowed to use an AED?' Regarding the skills assessment, the results showed that out of 94 participants (19.5%) with prior CPR training, only 8 of them were able to answer all the skills questions correctly. This could be explained by the fact that time is considered to be one of the factors that affect the competency and retention of CPR skills and knowledge, as most of the participants who have received previous CPR training (81%) did so more than two years ago.

Even though our study shows an obvious lack of knowledge and skills in BLS and CPR as well as an understanding of the role of an AED in cardiac arrest (only 64 teachers (16.7%) answered correctly), fortunately, most of the teachers (71.0%) have a positive attitude toward CPR, implying a willingness to get trained in CPR for different reasons. Several local and international studies support our results. For example, in Saudi Arabia, Al Enizi et al. conducted a cross-sectional study in the Qassim region in 2015, where they stated that secondary school teachers lack CPR training and hence have little knowledge or skills [13]. Alhejaili et al. reported similar results; hence, the findings are comparable [14]. Internationally, a study in Turkey revealed a lack of knowledge and skills in BLS among teachers [15]. In addition, another study in Shanghai, China, had a similar result with striction on the urgent need to educate staff members regarding first aid practices [16].

Although it has been established in Saudi Arabia that every school must assign a health guide (health educator), it has yet to be fully implemented. For this reason, we emphasize the ILCOR recommendations and the necessity of including them in the curriculum and making them mandatory for teachers [11]. It is obvious that teachers need more training courses to help them practice and learn first aid fundamentals, and our study reveals that they are willing to train and help. Furthermore, even though most of the teachers (372, 77.2%) wanted free CPR courses, the cost was not an issue, as only 10 (3.6%) of the teachers stated that cost was the reason they did not get previous CPR training. Meanwhile, 132 (34.0%) did not know where to attend a course, which reflects the importance of announcing and advertising such courses in schools. In addition, we believe this training should be periodic, as many studies state that the retention of CPR knowledge and skills can decrease dramatically over time. Binkhorst et al. stated that knowledge is retained better than skills, and with infrequent use of BLS, the skills usually worsen within three to six months after training, while knowledge is retained longer [17]. Another study by Nori et al. aimed to assess how often CPR training for nurses is necessary and revealed that the two-year retention of nurses' CPR knowledge and skills after CPR training courses decreased from 80.6% to 64.3% [18]. Additionally, the presence of a specialized health provider who is competent, prepared, and up-to-date on the emergency life-saving procedure is crucial to ensuring effective CPR in cardiopulmonary emergency events.

In sum, in this study, we emphasize the necessity and importance of delivering more BLS courses to schoolteachers and providing them with more opportunities to take the course. Also, healthcare professionals should take an active role in training and educating teachers on the fundamentals of first aid and BLS so they can take life-saving actions and be prepared to respond properly as first responders during emergencies. Also, since many studies show that skills retention is universally poor, our study conveys that it is salient to repeat the training courses every two years to retain the quality of the trainee's knowledge and skills level and overcome their knowledge gaps. Furthermore, the presence of a specialized health provider who is competent, prepared, and up-to-date on the emergency life-saving procedure is crucial to ensuring effective CPR in cardiopulmonary emergency events.

Although we tried to expand the generalizability of our study by taking a major city (Buraidah) and two smaller governorates (Uyun AlJiwa and Asyah), the study site selection could be a limitation for this study. Our greatest challenges remain the education of the lay rescuer and understanding and overcoming the barriers that prevent even trained rescuers from performing high-quality CPR [19]. 

## Conclusions

Along with several local and international studies supporting our results, this study reveals that although schoolteachers have a positive attitude toward training and a desire to help, their BLS knowledge and skills are poor and need improvement. For this reason, we accentuate the need to deliver more BLS courses to schoolteachers by announcing such courses in schools; thus, they know where and how to get their training provided by healthcare professionals. We emphasize the inclusion of ILCOR recommendations that necessitate the mandatory presence of BLS in the curriculum. Additionally, the training should be periodic to help retain the quality of the trainee's knowledge and skills level and overcome knowledge gaps.
